# DDX3X Induces Primary EGFR-TKI Resistance Based on Intratumor Heterogeneity in Lung Cancer Cells Harboring EGFR-Activating Mutations

**DOI:** 10.1371/journal.pone.0111019

**Published:** 2014-10-24

**Authors:** Koichiro Nozaki, Hiroshi Kagamu, Satoshi Shoji, Natsue Igarashi, Aya Ohtsubo, Masaaki Okajima, Satoru Miura, Satoshi Watanabe, Hirohisa Yoshizawa, Ichiei Narita

**Affiliations:** 1 Division of Respiratory Medicine, Department of Homeostatic Regulation and Development, Graduate School of Medical and Dental Sciences, Niigata University, Niigata, Japan; 2 Bioscience Medical Research Center, Niigata University Medical and Dental Hospital, Niigata, Japan; University of Parma, Italy

## Abstract

The specific mechanisms how lung cancer cells harboring epidermal growth factor receptor (EGFR) activating mutations can survive treatment with EGFR-tyrosine kinase inhibitors (TKIs) until they eventually acquire treatment-resistance genetic mutations are unclear. The phenotypic diversity of cancer cells caused by genetic or epigenetic alterations (intratumor heterogeneity) confers treatment failure and may foster tumor evolution through Darwinian selection. Recently, we found DDX3X as the protein that was preferentially expressed in murine melanoma with cancer stem cell (CSC)-like phenotypes by proteome analysis. In this study, we transfected PC9, human lung cancer cells harboring EGFR exon19 deletion, with cDNA encoding DDX3X and found that DDX3X, an ATP-dependent RNA helicase, induced CSC-like phenotypes and the epithelial-mesenchymal transition (EMT) accompanied with loss of sensitivity to EGFR-TKI. DDX3X expression was associated with upregulation of Sox2 and increase of cancer cells exhibiting CSC-like phenotypes, such as anchorage-independent proliferation, strong expression of CD44, and aldehyde dehydrogenase (ALDH). The EMT with switching from E-cadherin to N-cadherin was also facilitated by DDX3X. Either ligand-independent or ligand-induced EGFR phosphorylation was inhibited in lung cancer cells that strongly expressed DDX3X. Lack of EGFR signal addiction resulted in resistance to EGFR-TKI. Moreover, we found a small nonadherent subpopulation that strongly expressed DDX3X accompanied by the same stem cell-like properties and the EMT in parental PC9 cells. The unique subpopulation lacked EGFR signaling and was highly resistant to EGFR-TKI. In conclusion, our data indicate that DDX3X may play a critical role for inducing phenotypic diversity, and that treatment targeting DDX3X may overcome primary resistance to EGFR-TKI resulting from intratumor heterogeneity.

## Introduction

Treatments targeting signal addiction caused by oncogenic driver mutation have led to unprecedented results in the clinical setting. The use of epidermal growth factor receptor (EGFR)-tyrosine kinase inhibitors (TKIs) has significantly improved progression-free survival in lung cancer patients harboring activating EGFR mutations; however, it is still difficult to achieve a cure for lung cancer, particularly in patients with advanced-stage disease [1], [2]. The phenotypic diversity of cancer cells is based on both genetic and nongenetic factors and results in the survival of treatment-resistant cells. Indeed, most acquired resistance reflects the selection of cancer cells harboring stochastic resistance-conferring genetic alterations. However, the mechanisms through which cancer cells survive until acquisition of additional mutations are unclear. Sharma et al. demonstrated that a small subpopulation of reversibly drug-tolerant cells existed in all examined cancer cells and that drug-tolerant cells behaved as mother cells, giving rise to drug-resistant cells harboring additional mutations [3].

DEAD/H (Asp-Glu-Ala-Asp/His) box polypeptide 3, X-linked (DDX3X) is a member of the DEAD-box family of ATP-dependent RNA helicases and is located on the X chromosome [4]. DEAD-box helicases have multiple functions, including RNA splicing, mRNA export, transcriptional and translational regulation, RNA decay, ribosome biogenesis, and miRNA regulation [5], [6]. Thus, DDX3X is thought to be involved in the epigenetic regulation of gene expression. Our previous proteome analyses identified DDX3X as a protein preferentially expressed in purified CD133^+^ B16 melanoma cells, which possessed cancer stem cell (CSC)-like properties [7], [8]. Although DDX3X was originally reported to suppress tumor growth by modulating *p21^waf/cip1^* gene expression [9], DDX3X has also been shown to be directly correlated with oncogenesis [10], [11]. Recently, whole-exome sequencing identified DDX3X as a target of driver gene mutations that mediate pathogenic β-catenin signaling in medulloblastoma, which supports the CSC theory [12]–[16].

In this study, we sought to investigate the role of DDX3X in conferring EGFR-TKI resistance in lung cancer cells. Our data suggested that DDX3X may represent a novel therapeutic target for overcoming intratumor heterogeneity in lung cancer patients harboring EGFR-activating mutations.

## Materials and Methods

### Tumor cells

PC9 cells lung adenocarcinoma cells harboring an EGFR exon 19 deletion mutation, were provided from Riken BioResource Center and maintained in culture medium (CM) containing RPMI 1640 medium supplemented with 10% heat-inactivated lipopolysaccharide (LPS)-qualified fetal calf serum (FCS), 0.1 mM nonessential amino acids, 1 µM sodium pyruvate, 100 U/mL penicillin, and 100 µg/mL streptomycin sulfate (all from Life Technologies, Inc., Tokyo, Japan). HCC4006 lung adenocarcinoma cells harboring an EGFR exon 19 deletion mutation were purchased from American Type Culture Collection and were cultured in CM.

### Transfection of PC9 cells with *DDX3X* cDNA

Transfection of lung cancer cells with *DDX3X* cDNA was performed using a Myc-FLAG-tagged open reading frame (ORF) clone of human DDX3X transcript variant 1 as transfection-ready DNA (Origene Technologies, Inc., Rockville, MD, USA) according to the manufacturer’s protocol. Experimental cells were incubated with fresh medium containing G418 (600 µg/mL, Promega, Madison, WI, USA), and the medium was replaced with fresh G418-containing medium every 3–4 days until resistant colonies were identified.

### Knockdown of DDX3X by shRNA

Knockdown of DDX3X was performed using an shRNA lentiviral (pLKO.1-puro) plasmid (Sigma-Aldrich, St. Louis, MO, USA) having the following DDX3X target sequence: 5′-CCGGACGTTCTAAGAGCAGTCGATTCTCGAGAATCGACTGCTCTTAGAACGTTTTTTG. PC9 cells were plated in fresh medium, and hexadimethrine bromide (8 µg/mL) was added to each well. The cells were cotransfected with the pLKO.1-puro plasmid plus the packaging vector according to the manufacturer’s protocol. The medium was changed approximately 16 h after transfection, and the cells were cultured for an additional 48–72 h. Experimental cells were incubated with fresh medium containing puromycin (2.0 µg/mL), and the medium was replaced with fresh puromycin-containing medium every 3–4 days until resistant colonies were identified. A minimum of five puromycin-resistant colonies were picked, and each clone was expanded for the assay. The efficiency of DDX3X knockdown was determined by immunoblotting.

### Monoclonal antibodies (mAbs) and flow cytometry

PE-conjugated anti-CD44 (IM7), anti-E-cadherin (67A4), and anti-vimentin (GF2); fluorescein isothiocyanate (FITC)-conjugated anti-N-cadherin (8C11) were purchased from BD Pharmingen (San Diego, CA, USA). Analysis of cell-surface phenotypes was conducted through direct immunofluorescent staining of 0.5–1×10^6^ cells with the above-mentioned fluorophore-conjugated mAbs and then treated with 1% paraformaldehyde. In each sample, 1×10^4^ cells were analyzed using a FACScalibur flow microfluorometer (Becton Dickinson, Sunnyvale, CA, USA). PE-conjugated subclass-matched antibodies used as isotype controls were also purchased from BD Pharmingen. The samples were analyzed with CellQuest software (BD).

### Immunoblotting assay

Cells were harvested and lysed in Nonidet P-40 buffer containing a protease-inhibitor mixture (Sigma). Equal amounts of protein were subjected to sodium dodecyl sulfate polyacrylamide gel electrophoresis (SDS-PAGE) on Mini-PROTEAN TGX Any kD Precast Gels (BioRad, Hercules, CA, USA) and subsequently transferred to polyvinylidene difluoride membranes (Millipore, Billerica, MA, USA). Immunoblots from tumor cell lysates were probed with antibodies against DDX3X (Sigma), EGFR, phospho-EGFR (Tyr1068), phospho-EGFR (Tyr1173), phospho-EGFR (Tyr845), Akt, phospho-Akt, Erk1/2, phospho-Erk1/2, and β-actin (Sigma). All antibodies except for anti-DDX3X and anti-β-actin were purchased from Cell Signaling Technology Inc. (Danvers, MA, USA). Secondary antibodies consisted of anti-mouse IgG (BioRad) and anti-rabbit IgG conjugated to horseradish peroxidase (Abcam, Cambridge, MA, USA). Immunoreactive protein bands were visualized using an ECL kit (Pierce). At least three independent experiments were performed for all analyses.

### Aldefluor assays

ALDH activity was detected using the ALDEFLUOR kit (StemCell Technologies Inc., Vancouver, Canada) according to the manufacturer’s instructions. Briefly, Aldefluor assays were carried out by suspending cells in Aldefluor assay buffer containing 1.5 µM bodipy-aminoacetaldehyde, an ALDH substrate. Cells were then incubated for 30 min at 37°C and analyzed with a microfluorometer.

### Cell viability assays

A total of 5×10^4^ tumor cells were seeded in 24-well plates on day 0. Twenty-four hours after seeding, erlotinib or vehicle was added. Numbers of live and dead cells were counted with trypan blue staining. % Survival indicates a percentage of live cells, which excluded trypan blue staining, based on total cell number in a well. Samples were prepared in triplicate.

### MTT assays

Tumor cells were seeded at 5×10^3^ cells per well in flat-bottomed 96-well culture plates and cultured in CM. Following a 24-h incubation period to allow them attach to culture plates, cells were treated with erlotinib for 48 h. The culture plates were centrifuged for nonadherent tumor cells and the medium was removed as much as possible and replaced with 100 µl of fresh medium. The 3-(4,5-dimethylthiazol-2-yl)-2,5-diphenyltetrazolium bromide (MTT) assay for cell viability was performed by the addition of 10 µL MTT reagent per well for 2 h at 37°C, followed by the addition of 100 µL/well dye-solubilizing detergent reagent. The supernatant was aspirated and absorbance was measured at 570 nm. Results are representative of three independent experiments.

### LEF/TCF Reporter Assays

PC9 cells were transiently transfected with the lentivirus vector including TCF/LEF *Firefly* luciferase reporter construct and internal control *Renilla* luciferase construct, or non-inducible *Firefly* luciferase reporter construct and internal control *Renilla* luciferase construct (Cignal TCF/LEF Reporter kit, Qiagen). Cells were transfected with Lipofectamine 2000 and selected for 2 days in puromicin. Transfected cell were then reseeded into 24 well plates at 30,000 cells/well before treatment with recombinant Wnt3a (100 ng/ml; R&D Systems) for 12 hr. Dual Luciferase reporter assay was performed according to the manufacturer’s instructions (Dual-Luciferase Reporter Assay System, Promega), and promoter activity values are expressed as relative light units. Relative light units were calculated as *Firefly*-luciferase/*Renilla*-luciferase. Experiments were done in triplicates.

### Statistical analysis

Unless otherwise indicated, statistical analyses were performed using GraphPad Prism 6.0 software (GraphPad Software, Inc., La Jolla, CA, USA) and unpaired two-tailed Student’s *t* tests. For comparisons among more than three groups, two-way analysis of variance (ANOVA) with Bonferroni’s post-test was used. Differences with *P*-values of less than 0.05 were considered significant. The results of experiments are presented as arithmetic means ± SDs.

## Results

### DDX3X overexpression induces stem cell-like phenotypes

**Figure 1:**
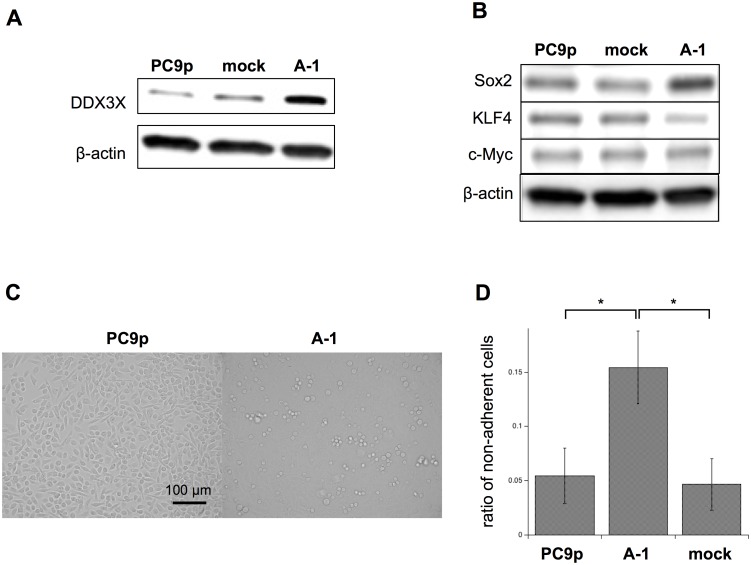
**A.** Immunoblotting analysis of DDX3X in parental PC9 cells, mock transfectants, and A-1 cells that were transfected with cDNA encoding DDX3X. **B.** Immunoblotting analysis of Sox2, KLF4, and c-Myc, in parental PC9 cells, mock transfectants, and A-1 cells. **C.** Analysis of anchorage-independent cell proliferation by phase-contrast microscopy. **D.** 1×10^5^ PC9 parental cells or A-1 cells were cultured on 24-well plates for 3 days. Nonadherent cells were collected by gentle rocking in culture medium. The ratio of nonadherent cells was calculated based on the total number of adherent cells. Dead cells were excluded with trypan blue exclusion method. *P<0.05. Data are presented as the mean ± SD of three independent experiments.

We recently reported that DDX3X expression is upregulated in the CD133^+^ melanoma subpopulation, which possesses CSC-like properties, such as tumor sphere formation and high tumorigenicity [8]. To elucidate whether DDX3X expression was correlated with CSC-like properties, we transfected PC9 cells, lung adenocarcinoma cells harboring an EGFR exon 19 deletion mutation, with cDNA encoding DDX3X and established a novel cell line, termed A-1 cells, which overexpressed DDX3X (Fig. 1A). As depicted in Fig. 1B, DDX3X overexpression resulted in upregulation of Sox2, a Yamanaka transcriptional factor that induces pluripotent stem cells. Moreover, the expression of Snail, a DDX3X-regulated transcription factor that promotes cell migration and metastasis in many types of cancers [17], seemed to be upregulated in A-1 cells (Fig. S1A). Additionally, we observed a significant increase in the number of cells proliferating in an anchorage-independent manner (Fig. 1C and 1D).

### DDX3X mediated EGFR-TKI resistance

**Figure 2:**
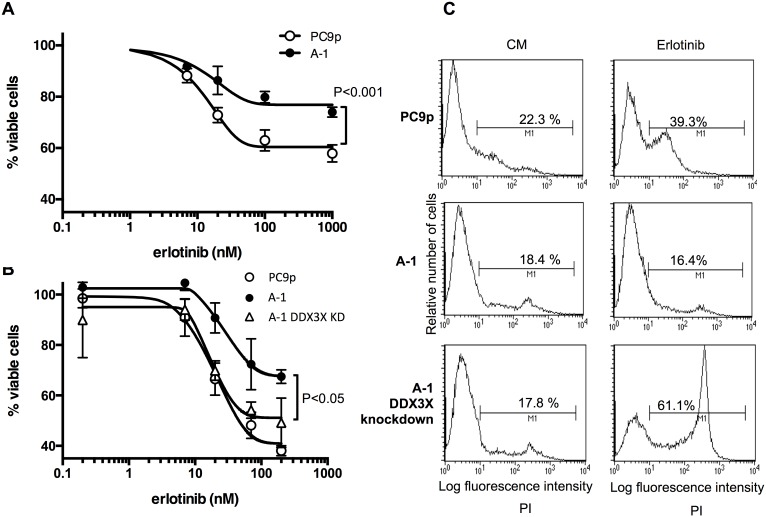
**A.** MTT assay of parental PC9 cells and A-1 cells. Cancer cells were treated with the indicated concentration of erlotinib for 48 h. Samples were prepared in triplicate. Data are presented as the mean ± SD. **B.** MTT assay of parental PC9 cells, A-1 cells, and A-1 cells with knockdown of DDX3X. Cancer cells were treated with the indicated concentration of erlotinib for 48 h. Samples were prepared in triplicate. Data are presented as the mean ± SD. **C.** Flow cytometry analysis of cancer cells treated with 20 nM erlotinib for 72 h.

**Figure 3:**
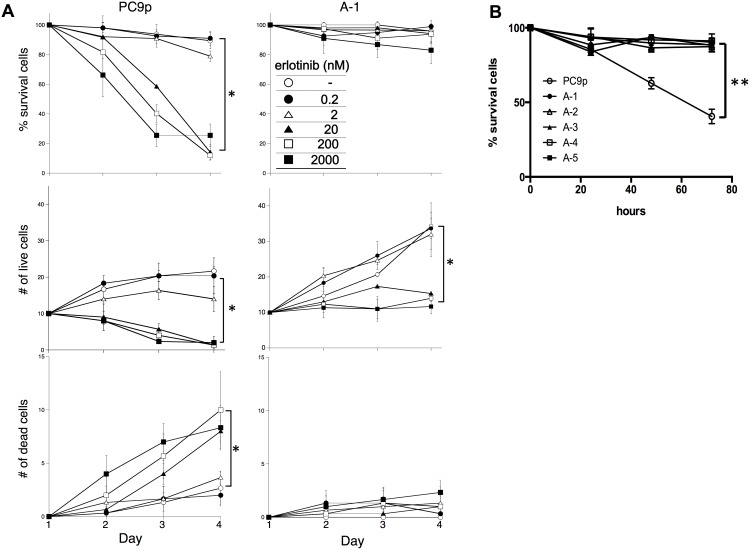
**A.** A total of 5×10^4^ tumor cells were seeded in 24-well plates on day 0. Twenty-four hours after seeding, erlotinib or vehicle was added at the indicated concentrations. Numbers of live and dead cells were counted with trypan blue staining. % Survival indicates a percentage of live cells, which excluded trypan blue staining, based on total cell number in a well. Samples were prepared in triplicate. Data are presented as the mean ± SD. * is used to indicate significance. **B.** The %Survival of cancer cells treated with 20 nM erlotinib was demonstrated. A-1, 2, 3, 4, and 5 indicate clone numbers that were transfected with cDNA encoding DDX3X. The %Survival indicates the percentage of live cells that excluded the trypan blue stain, based on total cell number in a well. Samples were prepared in triplicate. Data are presented as the mean ± SD. **p<0.001.

Since stem cells utilize stem cell-specific signaling; therefore, CSC-like phenotypes may be associated with the activation of different signaling pathways other than EGFR signaling resulting in alteration of sensitivity to EGFR-TKI. Thus, next we examined EGFR-TKI sensitivity of A-1 cells. As shown in Fig. 2A, a significant resistance to 48-h treatment with the EGFR-TKI erlotinib was observed in A-1 cells. Knockdown of DDX3X restored sensitivity to EGFR-TKI (Fig. 2B). Although decrease of % viable cells was observed in A-1 cells when cells were treated with erlotinib at concentration greater than 20 nM, it was not clear if cells were dying or not. To test whether A-1 cells were exhibiting reduced viability or simply reduced proliferation following 72-h erlotinib treatment, dead cells were stained with propidium iodide (PI) and analyzed with a microfluorometer. While the number of dead cells increased in the parental PC9 cells, no increase was detected in A-1 cells (Fig. 2C). This phenomenon was confirmed by counting cell numbers for three days using the trypan blue exclusion method (Fig. 3A). The number of dead PC9 cells was significantly increased in the presence of erlotinib at concentrations greater than 20 nM. In contrast, A-1 cells stopped proliferating when exposed to erlotinib at concentrations greater than 20 nM, but dead A-1 cell numbers did not differ from those in untreated cells, even after three days of exposure to 2000 nM erlotinib. All other clones that were engineered to overexpress DDX3X exhibited the same resistance to erlotinib as A-1 cells (Fig. 3B).

### DDX3X reduced EGFR signaling in cancer cells harboring EGFR-activating mutations

**Figure 4:**
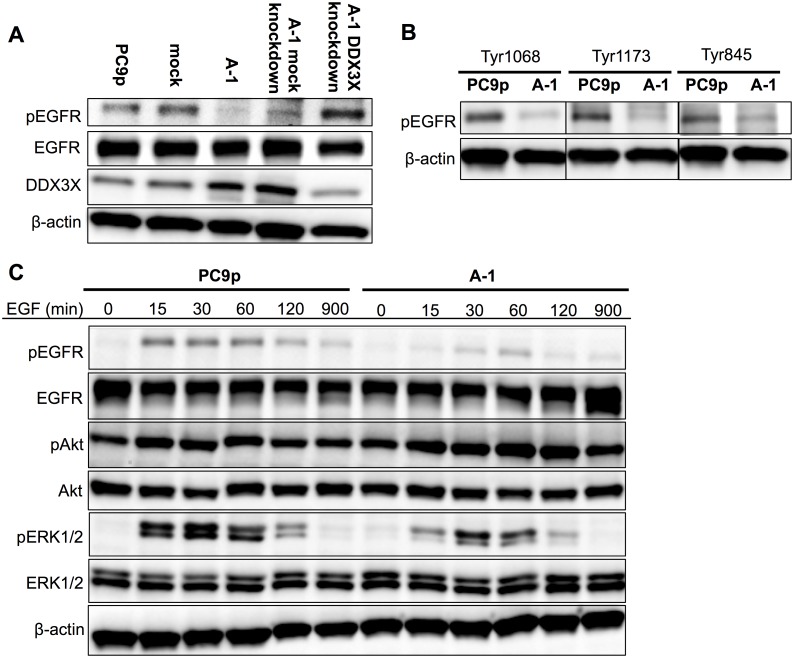
**A.** Immunoblotting analysis of EGFR, phospho-EGFR (Tyr1068), and DDX3X in cancer cells without EGF supplementation. **B.** Immunoblotting analysis of phospho-EGFR at Tyr1068, Tyr1173, and Tyr845 in parental PC9 cells and A-1 cells. **C.** Kinetic analysis of EGFR signaling in parental PC9 cells and A-1 cells. EGFR, phospho-EGFR (Tyr1068), Akt, phospho-Akt, ERK1/2, and phospho-ERK1/2 levels were analyzed by immunoblotting at 0, 15, 30, 60, 120, and 900 min after the addition of 100 ng/mL EGF.

The survival and proliferation of PC9 cells are dependent on an EGFR signaling addiction because PC9 cells possess driver mutations in the *EGFR* gene that enhance tyrosine kinase activity [18], [19]. Thus, we analyzed whether EGFR signaling was affected by DDX3X overexpression. Consistent with previous reports, Tyr1068 of EGFR was phosphorylated in parental PC9 cells and mock transfectants, even in the absence of EGF (Fig. 4A); however, almost no EGFR phosphorylation was detected in A-1 cells. Additionally, knockdown of DDX3X in A-1 cells restored EGFR phosphorylation. To confirm that this phenomenon was not limited to PC9 cell line, we transfected HCC4006, a human lung adenocarcinoma harboring an EGFR exon 19 deletion, with cDNA encoding DDX3X (HCC4006 S1, HCC4006 S2). HCC4006 S1 and S2 also lost EGFR phosphorylation (Fig. S1B). EGFR possesses multiple Tyr residues that undergo phosphorylation. For example, the GRB2 adaptor protein binds activated EGFR at phosphorylated Tyr1068 [20], and phosphorylated Tyr1173 provides a docking site for the Shc scaffold protein, which is involved in mitogen-activated protein kinase (MAPK) signaling activation [21]. Additionally, Tyr845 can be phosphorylated by other kinases, including Src, and has been shown to be phosphorylated in many gefitinib-resistant EGFR mutants [22]–[24]. Therefore, we next examined the phosphorylation states of these three tyrosine residues (Tyr845, Tyr1068, and Tyr1173) in the presence of EGF. Interestingly, we found reduced phosphorylation of all examined tyrosine residues in EGFR in A-1 cells (Fig. 4B).

In the presence of 100 ng/mL EGF, EGFR phosphorylation (Tyr1068) was increased in parental PC9 cells (Fig. 4C). In contrast, ligand-induced EGFR phosphorylation and subsequent extracellular signal-regulated kinase (ERK) phosphorylation were significantly attenuated in A-1 cells.

In addition to being regulated by phosphorylation, EGFR can generate signaling through phosphorylation of other human epidermal growth factor receptor (HER) family proteins by forming heterodimers. Crosstalk of Met with the EGFR signaling pathway has also been shown to be involved in EGFR-TKI resistance [25]. Thus, we tested whether HER2, HER3, HER4, and Met were phosphorylated. However, no differences in the phosphorylation states of HER2 or Met were found between parental PC9 cells and A-1 cells (Fig. S1C, D). Additionally, HER3 and HER4 were not phosphorylated in these cells (data not shown).

### A small subpopulation of PC9 cells strongly expressed DDX3X and exhibited CSC-like phenotypes

**Figure 5:**
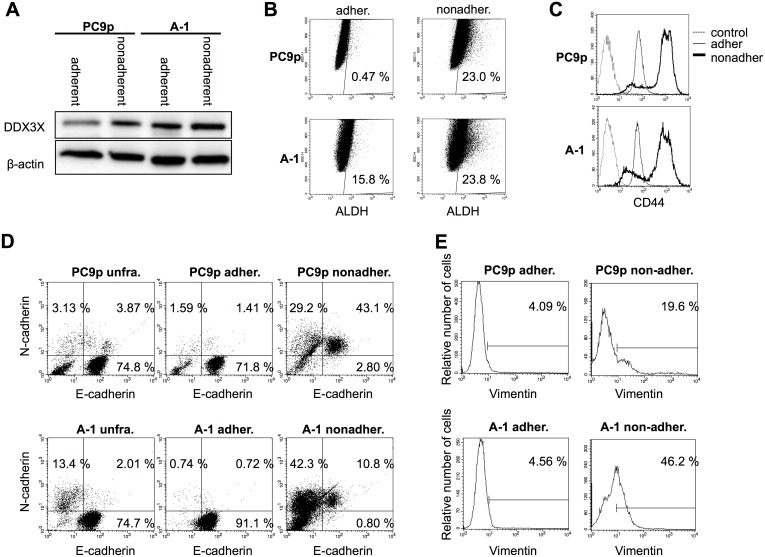
**A.** Immunoblotting analysis of DDX3X expression in adherent and nonadherent parental PC9 cells and A-1 cells. **B.** ALDEFLUOR assays were carried out by suspending cells in Aldefluor assay buffer containing 1.5 µM bodipy-aminoacetaldehyde, an ALDH substrate. Cells were then incubated for 45 min at 37°C and analyzed according to the manufacturer’s instructions. **C.** Flow cytometry analysis of adherent and nonadherent parental PC9 cells and A-1 cells. Histgrams indicate CD44 expression. Dotted lines indicate isotype control, thin lines indicate adherent cells, and the thick lines indicate nonadherent cells. **D.** Flow cytometry analysis of unfractionated, adherent, and nonadherent cells derived from parental PC9 cells and A-1 cells. X-axis indicates fluorescence intensity of E-cadherin, and Y-axis indicates fluorescence intensity of N-cadherin. **E.** Flow cytometry analysis of nonadherent cells isolated from parental PC9 and A-1 cells. Histogram plots show vimentin expression on gated N-cadherin+ cells.

Accumulating evidence has demonstrated that almost all tumor cells display remarkable variability in phenotypes based on genetic and epigenetic heterogeneity [26]. If DDX3X indeed plays a role in inducing a stem cell-like state and signal switching, a minor population of DDX3X-overexpressing parental PC9 cells may exist. Therefore, since A-1 cells exhibited the tendency to proliferate in an anchorage-independent manner, we examined a minor subpopulation of nonadherent PC9 cells. Surprisingly, this nonadherent population of parental PC9 cells exhibited almost the same level of DDX3X expression as A-1 cells (Fig. 5A). We examined the expression of ALDH, a putative CSC marker (Fig. 5B). Although only 0.47% of adherent PC9 cells expressed ALDH, 23.0% of nonadherent cells exhibited ALDH activity. On the other hand, 15.8% of adherent A-1 cells and 23.8% of nonadherent A-1 cells were ALDEFLUOR positive. It is likely that ALDEFLUOR-positive cells exist only in the cell population that strongly express DDX3X. Moreover, the majority of nonadherent cells but not adherent cells exhibited strong expression of CD44, another putative CSC marker (Fig. 5C).

It was demonstrated that during EMT, E-cadherin is downregulated while neural cadherin (N-cadherin) is upregulated, referred to as cadherin switch and that cadherin switch promotes cancer invasion and metastasis via increasing cancer cell motility [27]–[30]. Thus, we examined whether E/N cadherin switch was induced by DDX3X. As shown in Fig. 5D, 15.5% of unfractionated A-1 cells and 7.0% of unfractionated parental PC9 cells expressed N-cadherin, thus, it is likely that N-cadherin expression was promoted by DDX3X. Adherent cells were E-cadherin single positive and N-cadherin^+^ cells belonged to nonadherent cell population. Interestingly, 43.1% of nonadherent parental PC9 cells expressed both E-cadherin and N-cadherin and 29.2% was E-cadherin^−^N-cadherin^+^. It seems that incomplete cadherin switch occurred in parental PC9 cells. In contrast, 42.3% of nonadherent A-1 cells were N-cadherin single positive and 10.8% was E-cadherin^+^N-cadherin^+^. 21.7% of gated N-cadherin^+^ in parental PC9 cells, and 47.7% of gated N-cadherin^+^ in A-1 cells expressed vimentin (Fig. 5E). Taken together, a unique subpopulation that was undergoing EMT existed in nonadherent cells of both A-1 and parental PC9 and that DDX3X facilitated E/N cadherin switch followed by vimentin expression.

### Nonadherent cell population of parental PC9 lacked EGFR signaling and exhibited resistance to EGFR-TKI

**Figure 6:**
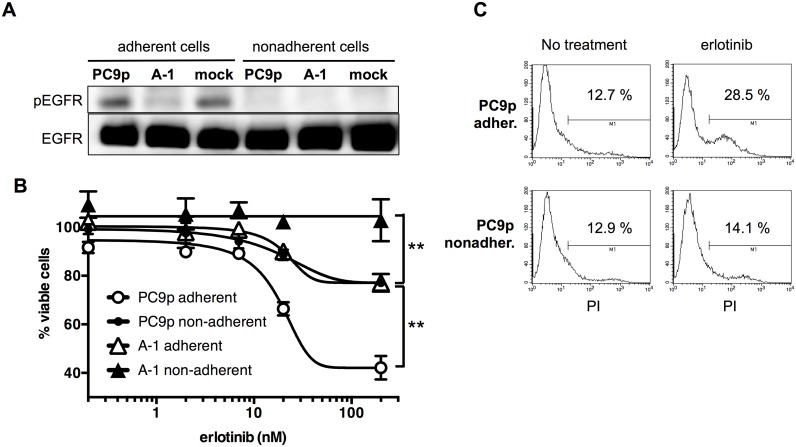
**A.** Immunoblotting analysis of EGFR and phospho-EGFR (Tyr1068) in adherent and nonadherent cancer cells. **B.** MTT assays were used to measure cell proliferation in adherent and nonadherent PC9 cells and A-1 cells following erlotinib treatment. Data are presented as the mean ± SD. Samples were prepared in triplicate. **P<0.001. **C.** Flow cytometry analysis with PI staining was used to examine dying cells.

**Figure 7:**
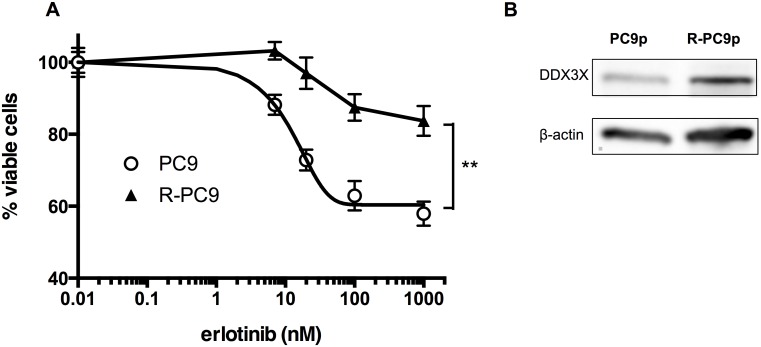
**A.** MTT assays were used to determine the proliferation of adherent and nonadherent PC9 cells, as well as R-PC9 cells, which were obtained by long-term exposure to increasing concentrations of erlotinib. Data are presented as the mean ± SD. Samples were prepared in triplicate. * indicates significant differences by two-way ANOVA and ad hoc analysis. **B.** Immunoblotting analysis of DDX3X expression in parental PC9 cells and R-PC9 cells.

As shown in Fig. 6A, all nonadherent cells lacked EGFR phosphorylation as A-1 cells. Moreover, the nonadherent population of parental PC9 cells was resistant to the EGFR-TKI erlotinib, similar to adherent A-1 cells (Fig. 6B). Because nonadherent cells may include dead cells, we examined the percentages of dying cells with PI staining. Fig. 6C shows that 28.5% of adherent parental PC9 cells were dying after 72-h erlotinib treatment. In contrast, the percentage of dying cells was only 14.1% in nonadherent PC9 cells. Next, we cultured parental PC9 cells in the presence of increasing concentrations of erlotinib (up to 3000 nM) to obtain resistant cells. After 3-month exposure to erlotinib, we established R-PC9 cells, which were able to survive in CM containing 3000 nM erlotinib. Fig. 7A demonstrates that R-PC9 cells were significantly resistant to erlotinib. R-PC9 cells exhibited strong expression of DDX3X (Fig. 7B). Collectively, our data demonstrated that parental PC9 cells contained a minor population exhibiting strong expression of DDX3X, CSC-like phenotypes, the EMT, and primary resistance to EGFR-TKI. The minor population can survive long treatment with EGFR-TKI.

### β-Catenin signaling induced by DDX3X

Although A-1 cells lacked EGFR phosphorylation and subsequent signaling, they showed almost the same proliferation rate as parental PC9 cells *in vitro*. It is likely that EGFR signaling is replaced by other signaling pathways. Stem cells utilize specific signaling pathways, such as Wnt/β-catenin or hedgehog, instead of receptor type tyrosine kinase signaling; moreover, a certain subset of CSC maintenance is dependent on Wnt/β-catenin signaling [31], [32]. DDX3X is required for Wnt/β-catenin signaling, and mutated DDX3X has been shown to play a role in pathogenic β-catenin signaling in medulloblastoma [13], [33]. Therefore, we next analyzed Wnt/β-catenin signaling. TCF/LEF luciferase reporter assay showed that β-catenin signaling was promoted in A-1 cells in the presence or absence of Wnt3a (Fig. S1E).

## Discussion

In this study, we investigated the role of DDX3X in acquisition of EGFR-TKI resistance in lung cancer cells. Our results revealed that cancer cells harboring EGFR-activating mutations could turn off EGFR signaling and lose EGFR signal addiction. Tyr residues in EGFR of the lung cancer cells overexpressing DDX3X were not phosphorylated even in the presence of EGF. This was an unexpected and surprising finding because EGFR-activating mutations cause the EGFR kinase domain to remain in the active conformation, even in the absence of ligands, resulting in the phosphorylation of tyrosine residues in the C-terminal tail segments. The precise mechanism through which DDX3X inhibits the phosphorylation of tyrosine residues in EGFR is unclear. However, inhibition of tyrosine kinase activity is unlikely to be involved because not only tyrosine residues subjected to autophosphorylation in C-terminal tail segments but also Tyr845 in the kinase domain, which can also be phosphorylated by Src, were not phosphorylated. The observation that DDX3X mediated Wnt/β-catenin signaling is consistent with a recent report describing the Wnt-dependent stimulation of casein kinase 1 and promotion of disheveled phosphorylation by DDX3X in mammalian cells [33]. Furthermore, whole-exome analyses identified DDX3X as a component of pathogenic β-catenin signaling, in Wnt subgroup medulloblastoma [12]–[14]. Taken together, our current data and these previous studies indicate that DDX3X is a critical component of the Wnt/β-catenin signaling pathway and that signal switching from EGFR addiction to β-catenin signaling could be induced by DDX3X in lung cancer cells harboring EGFR-activating mutations.

DDX3X is an RNA helicase that is highly conserved from yeast to human and modulate the structure of RNA [34]. Thus, RNA helicases are believed to participate in RNA transcription, splicing, and translation. Human DDX3 has two closely related genes, DDX3X and DDX3Y, which are encoded in the X and Y chromosomes, respectively. DDX3Y is uniquely expressed in germ cells and is essential for spermatogenesis [35]. Interestingly, we found that DDX3X induced increase of cancer cells accompanied by stem-like phenotypes such as upregulated expression of Sox2, ALDH, and CD44. Moreover, anchorage independent proliferation, cadherin switching from E-cadherin to N-cadherin, vimentin expression, was observed in these cells. Consistent with our findings, tumors harboring upregulated DDX3X have been reported to exhibit stem-like properties and/or evidence of the EMT in melanoma, breast cancer, and leukemia [8], [10], [12], [36]. Especially, medulloblastoma, in which DDX3X has been shown to act as a driver gene, is similar in appearance and differentiation potential to neural stem and progenitor cells. However, it is unique finding that untreated lung adenocarcinoma cells contained a subpopulation strongly expressing DDX3X and accompanied by signal switching, CSC-like phenotypes, and evidence of the EMT.

In summary, this study provided the first demonstration that DDX3X induced loss of EGFR signal addiction resulting in resistance to EGFR-TKI, accompanied by CSC-like properties and occurrence of the EMT in lung cancer cells harboring EGFR-activating mutation. Our results showed that inhibition of DDX3X may be a promising therapy for overcoming primary EGFR-TKI-resistance mechanisms based on intratumor heterogeneity and for achieving a cure in lung cancer patients harboring EGFR-activating mutations.

## Supporting Information

Figure S1 **A.** Immunoblotting analysis of Snail in parental PC9 cells, mock transfectants, and A-1 cells. **B.** Immunoblotting analysis of EGFR, phospho-EGFR (Tyr1068), in HCC4006 cells, DDX3X trasnfectants (HCC4006 S1, S2), and mock transfectants without EGF supplementation. **C.** Human phospho-receptor tyrosine kinase array (R&D systems) was carried out according to the manufacturer’s protocol. **D.** Immunoblotting analysis of Met and phospho-Met. **E.** TCF/LEF luciferase reporter assays were carried out to examine Wnt/β-catenin signaling activity. Tumor cells transiently transfected with TCF/LEF luciferase reporter constructs were treated with recombinant Wnt3a (100 ng/ml) for 12 hr. Promoter activity values are expressed as relative renilla luciferase units (RLU). Experiments were done in triplicates. The data represents 3 independent experiments. Data are presented as the mean ± SD.
(TIFF)
