# Supplementary figures and images for: DDX3X Induces Primary EGFR-TKI Resistance Based on Intratumor Heterogeneity in Lung Cancer Cells Harboring EGFR-Activating Mutations
Source: PLoS One. 2014 Oct 24;9(10):e111019. doi: 10.1371/journal.pone.0111019 (PMC4208809; doi:10.1371/journal.pone.0111019)

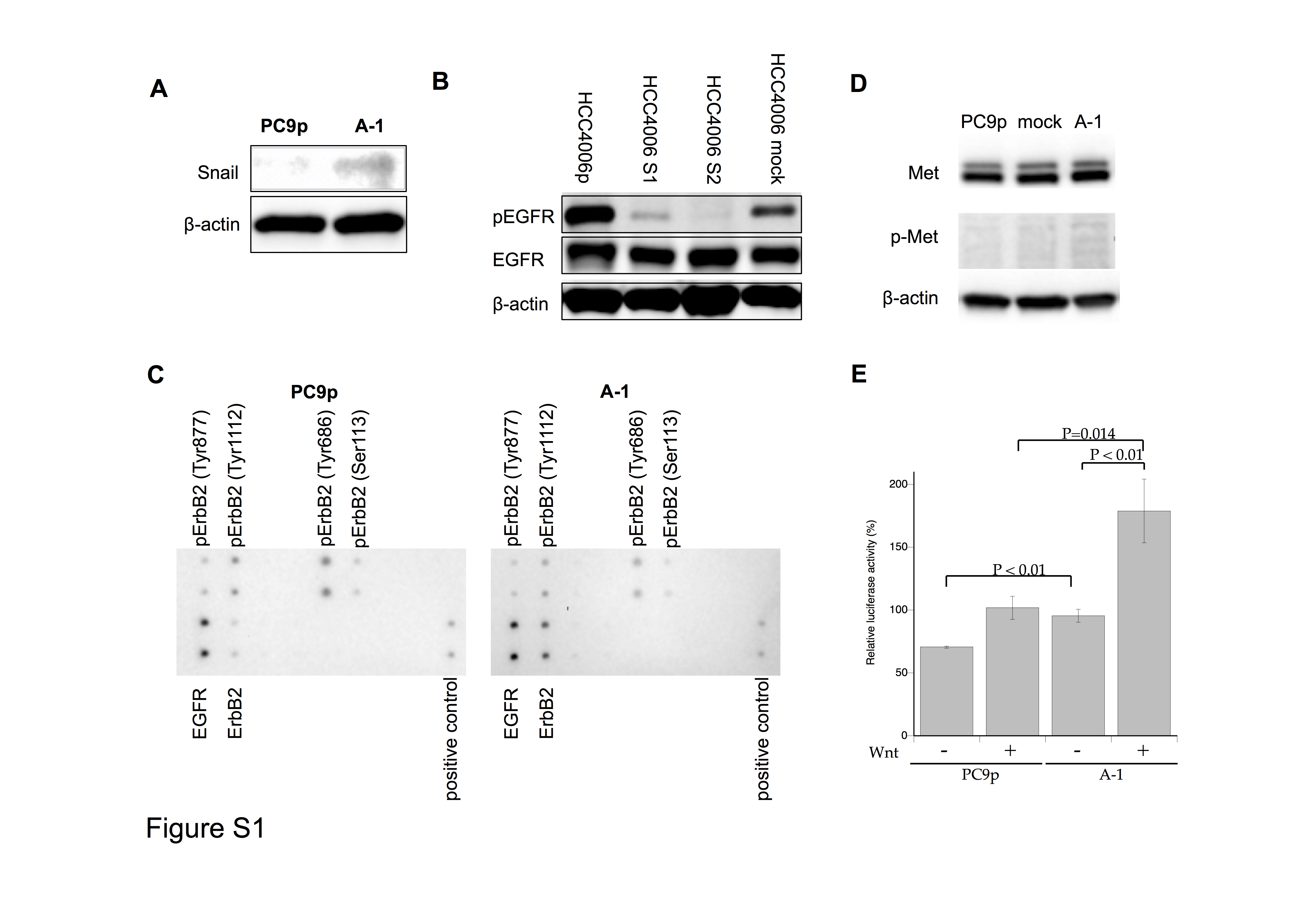

Supplement: Figure S1 — A. Immunoblotting analysis of Snail in parental PC9 cells, mock transfectants, and A-1 cells. B. Immunoblotting analysis of EGFR, phospho-EGFR (Tyr1068), in HCC4006 cells, DDX3X trasnfectants (HCC4006 S1, S2), and mock transfectants without EGF supplementation. C. Human phospho-receptor tyrosine kinase array (R&D systems) was carried out according to the manufacturer’s protocol. D. Immunoblotting analysis of Met and phospho-Met. E. TCF/LEF luciferase reporter assays were carried out to examine Wnt/β-catenin signaling activity. Tumor cells transiently transfected with TCF/LEF luciferase reporter constructs were treated with recombinant Wnt3a (100 ng/ml) for 12 hr. Promoter activity values are expressed as relative renilla luciferase units (RLU). Experiments were done in triplicates. The data represents 3 independent experiments. Data are presented as the mean ± SD. (TIFF) [file pone.0111019.s001.tiff]
